# Insights into Healthcare Professionals’ Perceptions and Attitudes toward Nanotechnological Device Application: What Is the Current Situation in Glioblastoma Research?

**DOI:** 10.3390/biomedicines11071854

**Published:** 2023-06-28

**Authors:** Federica Ragucci, Francesca Sireci, Francesco Cavallieri, Jessica Rossi, Giuseppe Biagini, Giovanni Tosi, Chiara Lucchi, Rodolfo Molina-Pena, Natalia Helen Ferreira, Mariana Zarur, Alba Ferreiros, William Bourgeois, François Berger, Miguel Abal, Audrey Rousseau, Frank Boury, Carmen Alvarez-Lorenzo, Emmanuel Garcion, Anna Pisanello, Giacomo Pavesi, Corrado Iaccarino, Luca Ghirotto, Maria Chiara Bassi, Franco Valzania

**Affiliations:** 1 Neurology Unit, Neuromotor and Rehabilitation Department, Azienda USL-IRCCS di Reggio Emilia, 42123 Reggio Emilia, Italy; 2 Clinical and Experimental Medicine PhD Program, University of Modena and Reggio Emilia, 41125 Modena, Italy; 3 Department of Biomedical, Metabolic and Neural Sciences, University of Modena and Reggio Emilia, 41125 Modena, Italy; 4 Department of Life Sciences, University of Modena and Reggio Emilia, 41125 Modena, Italy; 5 Inserm UMR 1307, CNRS UMR 6075, Université de Nantes, CRCI2NA, Université d’Angers, 49000 Angers, France; 6 Departamento de Farmacología, Farmacia y Tecnología Farmacéutica, I+D Farma (GI-1645), Facultad de Farmacia, and Health Research Institute of Santiago de Compostela (IDIS), Universidade de Santiago de Compostela, 15782 Santiago de Compostela, Spain; 7 Nasasbiotech, S.L., Canton Grande 9, 15003 A Coruña, Spain; 8 Braintech Lab, INSERM Unit 1205, Grenoble Alpes University, 38000 Grenoble, France; 9 Translational Medical Oncology Group (Oncomet), Health Research Institute of Santiago de Compostela (IDIS), University Hospital of Santiago de Compostela (SERGAS), 15706 Santiago de Compostela, Spain; 10 Département de Pathologie, CHU d’Angers, CRCINA Université de Nantes, 49933 Angers, France; 11 Département de Pathologie, CHU d’Angers, Université d’Angers, 49933 Angers, France; 12 Qualitative Research Unit, Azienda USL-IRCCS di Reggio Emilia, 42123 Reggio Emilia, Italy; 13 Medical Library, Azienda USL-IRCCS di Reggio Emilia, 42123 Reggio Emilia, Italy

**Keywords:** attitudes, awareness, bias, brain tumor, cancer, gliomas, healthcare professionals, nanotechnology, psychology

## Abstract

Nanotechnology application in cancer treatment is promising and is likely to quickly spread worldwide in the near future. To date, most scientific studies on nanomaterial development have focused on deepening the attitudes of end users and experts, leaving clinical practice implications unexplored. Neuro-oncology might be a promising field for the application of nanotechnologies, especially for malignant brain tumors with a low-survival rate such as glioblastoma (GBM). As to improving patients’ quality of life and life expectancy, innovative treatments are worth being explored. Indeed, it is important to explore clinicians’ intention to use experimental technologies in clinical practice. In the present study, we conducted an exploratory review of the literature about healthcare workers’ knowledge and personal opinions toward nanomedicine. Our search (i) gives evidence for disagreement between self-reported and factual knowledge about nanomedicine and (ii) suggests the internet and television as main sources of information about current trends in nanomedicine applications, over scientific journals and formal education. Current models of risk assessment suggest time-saving cognitive and affective shortcuts, i.e., heuristics support both laypeople and experts in the decision-making process under uncertainty, whereas they might be a source of error. Whether the knowledge is poor, heuristics are more likely to occur and thus clinicians’ opinions and perspectives toward new technologies might be biased.

## 1. Introduction

Glioblastoma (GBM) is the most common malignant tumor of the Central Nervous System (CNS) in adults accounting for 82% of cases of malignant glioma [1] and 45.6% of primary malignant brain tumors globally [2]. The annual incidence of gliomas is approximately 6 cases per 100,000 individuals worldwide [3,4]. The annual age-adjusted incidence of glioblastoma increases with age from 0.15 per 100,000 in children to a peak of 15.03 per 100,000 in patients aged 75–84 years [2,3]. Men are 1.6-fold more likely to be diagnosed with gliomas than women [4].

With the advent of the fifth edition of the WHO Classification of Tumors of the CNS, published in 2021, major changes in glioma classification have been introduced that advance the role of molecular diagnostics in glioma classification. Indeed, while the 2016 WHO classification distinguished between IDH-mutant and IDH-wildtype glioblastoma, in the current classification, all IDH-mutant diffuse astrocytic tumors are considered a single type (Astrocytoma, IDH-mutant) and are graded as CNS WHO grade 2, 3, or 4. IDH-wildtype glioblastoma should be diagnosed in the setting of an IDH-wildtype diffuse and astrocytic glioma if there is (1) microvascular proliferation, (2) necrosis, or (3) telomerase reverse transcriptase (TERT) promoter mutation, or (4) epidermal growth factor receptor (EGFR) gene amplification, or 5) +7/−10 chromosome copy number changes [5].

Brain tumor management is challenging. Immunological features of the CNS, such as the blood–brain barrier (BBB), may exclude most of the current therapies targeted to the brain [6] and urge the need for novel therapies. Nanotechnology is promising due to the ability of nanoparticles to navigate the BBB, ductility, and target precision [7].

A multidisciplinary specialized team is required to provide tailored health interventions since physical, neuro-cognitive, and emotional disruptions may threaten the outcome of patients with glioblastoma. Efforts to improve patients’ outcomes also rely upon efficacy in interaction, communication, and shared knowledge among healthcare team members [8,9,10]. When a new experimental therapy is about to be transferred from bench to bedside, a concern may arise among clinicians who might differ in background and literacy in novel therapeutic strategies such as nanocarrier-based tumor targeting. Moreover, individual perspectives toward the application of nanotechnologies might affect the willingness to adopt a new (experimental) practice in settings providing team-based care [11]. Therefore, professionals responsible for coordinating the workgroup and in a high decision-making role might be able to influence attitudes toward science and innovation in the healthcare structure itself.

## 2. Glioblastoma and Its Current Treatments

Even if a lot of progress in surgical, radiotherapeutic, and chemotherapeutic treatments has been made, almost all tumors recur, and salvage therapeutic options are limited with a poor overall prognosis [12,13]. Indeed, the current patient’s overall median survival is around 12–14 months [14] with a 5-year survival rate of 4–5%, and only a 26–33% survival rate at 2 years in clinical trials) [12].

Initial treatment of GBM starts with maximal safe resection that can provide a clinical benefit from a reduction in the mass effect [15]. Surgery currently aims to target the complete resection of the MRI T1w sequence-enhancing tumor and it is common knowledge that complete resection is associated with better survival than partial resection or biopsy [16]. However, as GBM relapses beyond the resected contrast-enhanced edges, it has recently been proposed to expand the surgical resection to the FLAIR-hyperintense area surrounding the contrast-enhancing region, obtaining the so-called supra marginal resection (SMR). In a study conducted by Vivas-Buitrago et al., SMR was associated with better overall survival (OS) in patients with IDH-wt GBM [17]. However, as the FLAIR region comprises both edematous functional brain parenchyma and infiltrative tumor cells, with a continuous reduction in tumor cell density toward the periphery, resection should balance oncological and neurological outcomes. Vivaz-Butraigo et al. reported a positive influence in SMR cases from 20% to 50%, with no clear advantage for higher resection [17,18].

Multiple randomized controlled trials have defined radiation therapy (RT) as a cornerstone of adjuvant treatment for newly diagnosed GBM after the surgical removal of the tumor for improving OS and progression-free survival (PFS) [19,20]. Due to the poor efficacy of many chemotherapies against GBM, the role of chemotherapy was considered controversial before the approval of temozolomide (TMZ) in 2005 [21]. Indeed, in 2005, a large, international, randomized, phase III trial demonstrated prolonged survival when daily TMZ chemotherapy (75 mg/m^2^ daily × 40–49 days) was added concomitantly to radiotherapy (60 Gy/30 fractions) followed by six cycles of maintenance temozolomide (150–200 mg/m^2^ × 5/28 days) [22]. Based on this landmark trial, TMZ/radiotherapy followed by the maintenance of TMZ has become the worldwide standard of care for patients with newly diagnosed glioblastoma [22,23,24]. TMZ is a lipophilic, monofunctional prodrug that belongs to the alkylating group known to arrest cell cycle at G2/M, and eventually lead to apoptosis. In subsequent years, many studies have reported that the combination of RT and TMZ improves even the long-term survival in glioma patients, confirming the superiority of the combination on a long-time basis compared to the RT alone [25]. Indeed, as reported in a large, randomized trial in GBM patients, the combination of RT and TMZ led to an overall survival of 9.8% (6.4–14.0) at 5 years, versus 1.9% (0.6–4.4) with radiotherapy alone (hazard ratio 0.6, 95% CI 0.5–0.7; *p* < 0.0001), confirming that the benefits of adjuvant temozolomide with radiotherapy lasted throughout the 5 years of follow-up [23]. O6-methylguanine-DNA methyl-transferase (MGMT) methylation status identifies patients most likely to benefit from the addition of temozolomide. It is well recognized today that MGMT methylation status identifies patients most likely to benefit from the addition of temozolomide [23]. As a result, MGMT methylation status has become the single most important prognostic factor in an era in which the vast majority of adults with glioma are treated with alkylating agent-based chemotherapy [4].

Afterward, bevacizumab, an anti-angiogenic drug, was approved as an adjuvant treatment for newly diagnosed GBM. However, a recent metanalysis confirmed that its use is associated with a longer PFS in adult patients with newly diagnosed glioblastoma, but with an inconsistent effect on OS [26]. The addition of tumor-treating fields (TTFields) to maintenance temozolomide chemotherapy for newly diagnosed glioblastoma patients has recently been incorporated as a new standard of care [24]. TTFields are generated via electrodes on the scalp with a unique array placement based on an individual’s MRI results and can disturb highly orchestrated dividing processes into GBM cells, sparing quiescent ones through its action on the microtubule assembly and the cleavage furrow [27]. In 2015, a randomized trial evaluated the efficacy and safety of TTFields used in combination with temozolomide maintenance treatment after chemoradiation therapy for patients with glioblastoma showing that adding TTFields to maintenance temozolomide chemotherapy significantly prolonged progression-free and overall survival [22].

Standard-of-care treatments for patients with recurrent glioblastoma are not well defined; treatment is selected on the basis of prior therapy, age, Karnofsky score (KPS), MGMT promoter methylation status, and patterns of disease progression [4]. Moreover, a significant proportion of patients may not even be eligible for second-line therapy [28]. Options include further surgical resection, reirradiation, systemic therapies such as lomustine, bevacizumab, or regorafenib, combined approaches, or supportive care alone [28]. A previous study assessed the association of clinical outcome with the extent of resection upon surgery for recurrent GBM showing that surgery at first recurrence improved outcome only if complete resection of contrast-enhancing tumor was achieved [29]. Re-irradiation (by using conventional radiotherapy, fractionated radiosurgery, or single fraction radiosurgery) has been proposed as a therapeutical option in patients with progressive GBM after the first adjuvant combined multimodality treatment [30]. Although the reported evidence in the literature uses different endpoints and outcome parameters, there is class III evidence that re-irradiation, especially in the form of stereotactic radiosurgery (SRS) and fractionated stereotactic radiosurgery (FSRS), can achieve tumor control on selected groups of patients, helping to maintain the neurological status, reduce steroid use, and in some cases, improve quality of life [30]. However, the majority of patients who are eligible for salvage therapy receive systemic treatment, mostly with nitrosourea-based regimens such as lomustine or bevacizumab [4]. In particular, lomustine has been increasingly used in clinical trials as a control arm, becoming the standard-of-care position in the setting of recurrent glioblastoma. Indeed, no other agent, with the possible exception of regorafenib, has shown superior activity to lomustine in recurrent glioblastoma [4]. However, as already reported for TMZ, the activity of lomustine is largely restricted to patients with tumors with MGMT promoter methylation [4]. Recently regorafenib, an oral multikinase inhibitor of angiogenic, stromal, and oncogenic receptor tyrosine kinases, has been evaluated in the treatment of recurrent glioblastoma in the REGOMA study (Regorafenib in Relapsed Glioblastoma), showing an encouraging overall survival benefit in recurrent glioblastoma [31]. Standard-of-care treatments for newly diagnosed and recurrent glioblastoma are summarized in Table 1.

## 3. Risk Perception of Nanotechnological Devices Application

Nano-related technologies are spreading fast across diverse fields of application (engineering, agri-food, computer science, medicine, and more), and regulatory, health, and safety uncertainty have already been explored [34,35]. While health, regulatory, and safety aspects have been investigated among end users and experts, clinicians’ risk perception related to nanotechnologies has been scarcely evaluated.

The very first theoretical model of risk perception is the psychometric paradigm [36,37], which draws a roadmap of potential factors affecting laypeople’s assessment of risk. According to this model, voluntary, more familiar, and less-impacting forecasted consequences of risks may affect the perception of the individual. Cognitive shortcuts (i.e., heuristics) [38,39] in judging risky operations are frequently used by people to reduce complexity in decision making, despite often being causes of systematic errors. Current emotional states and moral values [40,41] might also be the cause of the distortion of beliefs in front of uncertainty.

Cognitive, emotional, and moral biases in decision making under uncertainty are shared by both experts and laypeople [42]. Several exploratory studies have been conducted to understand laypeople’s behavior toward nanotechnologies [43,44,45]. As an example, the Nano-PAAF model [45] identifies three main factors that shape peoples’ risk assessment mental processes in evaluating nanotechnologies: cognitive (e.g., prior knowledge, attitude toward science and technology), affective (e.g., affective heuristics, fear, and uncertainty), and sociocultural (e.g., political and religious beliefs). According to this model, cognitive, affective, and sociocultural variables affect the risk–benefit trade-off of the individual with socio-demographic and contextual variables as moderators. Prior factual knowledge, awareness of nanotechnology, various sources of information, and confidence in science and technology may offset feelings of concern (affective heuristics) and support benefit perception. Indeed, in both experts and end users, factual knowledge and awareness about nanotechnology are associated with lower perceived risk and a more positive attitude toward its application. The product category itself might affect consumers’ attitudes toward nanotechnology, where food and medicine raise more concern than other fields of application [45,46].

With a focus on the experts, a greater need for information about safety and government regulation related to nanotechnology and its applications is claimed [47,48]. Organization members’ requests for government regulation relates to both the relevance of nanotechnology for the organization itself and the field of nanotechnology application or product category, as if physical contact with the product is expected [48].

In the last 10–20 years, clinical cancer therapy that integrates nanomedicine has been growing, and the use of nanotechnological devices in addressing tumor treatment is likely to be translated into a clinical application in the near future [49,50]. Neuro-oncology is one of the medical fields where the application of nanotechnologies might be helpful, especially if first-in-line treatments are missing, such as for recurrent glioblastoma. To date, nanotechnological devices supporting brain cancer therapy, namely high-grade gliomas, have not yet reached the post-market stage. Numerous clinical trials are running worldwide, ranging from early preclinical animal testing to phase III studies. Research has focused on describing clinicians’ attitudes from a scientific and regulatory perspective [51], while psychological aspects have been mostly overlooked. In fact, it is still unclear how cognitive and/or affective heuristics and personal ethics toward nanotechnology applied to the medical field interplay in risk assessment, especially among healthcare professionals. Personal beliefs toward nanotechnologies might affect both the risk perception and the intention to use, thus the probability of innovative treatments being actually introduced in clinical practice. As a contribution to a broader overview of the current state of research, we carried out an exploratory literature review through healthcare practitioners’ perceptions and intentions to use nanotechnology in the field of neuro-oncology.

## 4. Materials and Methods

### 4.1. Search Strategy and Eligibility Criteria

We conducted a first literature search of electronic databases (MEDLINE, Embase, Cinahl, Scopus, and Web of Science) focused on clinicians’ attitudes toward nanotechnology applications in glioblastoma ((“Glioblastoma”[Mesh]) OR “Brain Neoplasms”[Mesh] OR glioblastoma[title/abstract] OR brain tumor[title/abstract] OR brain cancer[title/abstract] OR brain neoplasm[title/abstract]) AND ((Nanotechnolog*[Title/Abstract] OR nanomedicine[Title/Abstract] OR “Nanomedicine”[Mesh] OR “Nanotechnology”[Mesh]) AND (Physicians OR Medical Residents OR Clinicians) AND (Perception OR Attitude OR Acceptance OR Knowledge)) which produced 18 records. Based on this search, we only identified studies focused on the biological properties of nanoparticles. On the grounds that literature on clinicians’ perspectives on nanotechnology within neuro-oncology was apparently missing, we considered smoothing our search criteria to collect as much information as possible on healthcare workers’ perspectives on nanotechnology and spot any possible piece of knowledge within glioblastoma.

Studies were included if healthcare workers’ knowledge (i.e., factual information) and/or opinions (i.e., judgment not necessarily based on facts) toward nanomedicine had been explored, regardless of the field of application. We did not include articles if qualitative or quantitative measurements had not been collected. Language, type of paper, nor publication date restriction was imposed.

**Figure 1:**
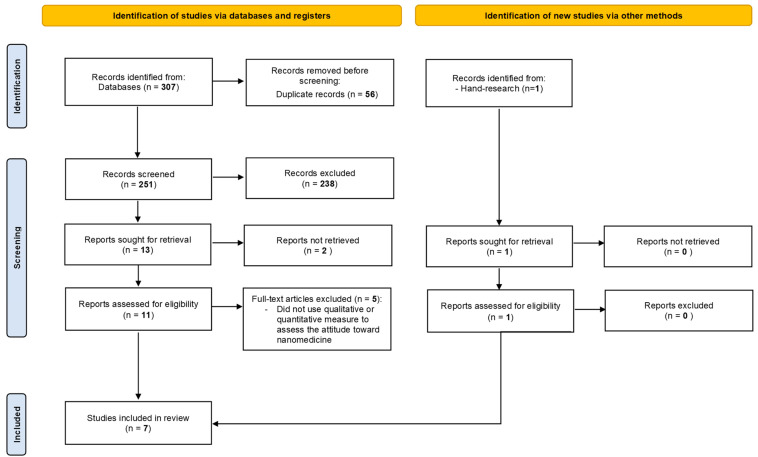
PRISMA flowchart.

Figure 1 illustrates the search process through the PRISMA flow diagram.

### 4.2. Study Selection

Studies had been identified by conducting a systematic search of electronic databases (MEDLINE, Embase, CINAHL, Scopus, and Web of Science) using keywords related to “nanotechnology” combined through the Boolean operator “AND” with keywords related to “perception” and “healthcare professionals” (see Table S2 for the full query). The search was performed by two independent reviewers (FR, MCB) for all articles published until 20 September 2022. Reference sections of included studies were checked. Discrepancies in the data extracted by the reviewers were solved by an independent author (FS).

### 4.3. Synthesis Analysis

We used a data extraction form to synthesize the most relevant information from the included studies (Supplementary Material Table S1). Professional profile, prior experience or education with nanomaterials (%), assessment measure used to collect data, reference to a theoretical background, and data analysis strategy guided discussion about current knowledge of healthcare professionals’ attitudes toward nanomedicine.

## 5. Results

The database search and additional hand research produced 252 eligible works net of duplicates. The abstract screening resulted in the exclusion of 238 records. The high amount of excluded records could be explained by the use of “loose-fitting” search keywords which could have included a large number of papers not relevant to the purposes of our research (e.g., articles focusing on medical or biological/biotechnological properties of nanodevices with any psychological perspective). Twelve full-text articles were retrieved for eligibility. Five full-text articles were excluded since they did not conduct a qualitative or quantitative assessment of healthcare professionals’ attitudes toward nanomedicine. Seven studies published from 2011 to 2021 set up in five countries were considered eligible, and thus appropriate for debating (Table 2).

### 5.1. Study Characteristics

Two studies in India [52,53], one study in Saudi Arabia [54], two studies in the United States of America [55,56], and two studies in Norway [57,58] were conducted.

Most studies explored knowledge and/or opinions about nanotechnology in nanomedicine as a whole, regardless of a specific field of application.

Cross-sectional study designs were used to assess students, medical residents, and clinicians’ opinions.

Study samples resulted in being heterogeneous, ranging from 23 to 851 healthcare workers aged between 18 to 70 years old. Perspectives on nanotechnology application in the medical field were collected by online or paper-and-pencil self-administered questionnaires from 8 to 35 items long. The most common measured constructs were intention to use, application awareness, and familiarity with nanomedicine or nanomaterials. Only one study [58] referred to a grounded theoretical measurement model, whereas most questionnaires were designed according to the authors’ personal preferences. Respondents were usually invited to express their agreement with a list of statements and/or questions from 2-point to 10-point rating scales. Results were reported descriptively as percentage agreement measures for a single item or a group of items underlying the same construct. Additional analyses were seldom performed to check the statistical significance of the results or to make inferences on the collected data.

### 5.2. Healthcare Professionals’ Attitude toward Nanomedicine

Participants attitudes toward nanomedicine were positive, although accurate knowledge of nanotechnology was poor (e.g., [54,56]). Inadequate access to information on nanotechnology was reported equally in studies conducted in 2011 and 2021. Only two studies [55,56] explored personal attitudes toward nanotechnology, meant as positive or negative feelings, and only one [56] supplied extra open-answer questions to ascertain practitioners’ knowledge about nanomedicine. Open-answer questions were used to assess responders’ self-perceived and factual knowledge of nanomedicine agreement. Data suggested that high-perceived knowledge did not find open-answer questions to be answered properly. For example, only a few upcoming clinicians could report the name of one drug-targeting tumor cell supported by nanomedicine.

Education gaps in nanomedicine with the need to update universities’ curricula was primarily demanded by students and medical residents [54,55,56] with about 80% of respondents who claimed more education on nanotechnology was needed. Similar evidence is documented among clinicians [54,57], with up to 90% of respondents expressing interest in accessing more information about nanomaterials. Nevertheless, self-reported literacy in nanomedicine (e.g., “Have you heard about nanotechnology?”) was heterogenous; two studies conducted in 2011 [54] and 2021 [53] reported a 50% mean percentage of “yes” responders, whilst one study conducted in 2019 [56] reported that 87% of participants had heard of nanotechnology. Media and television head the list of sources of information about nanotechnology, thereby overcoming scientific journals and formal education. One study pointed out that personal attitudes and subjectively perceived confidence toward nanomaterials would influence clinicians’ intention to use them [58]. Norwegian dentists and dental hygienists reported feeling safe to use nanomaterials in clinical practice if perceived benefits and risks were balanced [57]. Furthermore, knowledge was found to influence risk perceptions related to the use of nanomaterials [58], claiming a more in-depth exploration of socio-cognitive factors. On the other hand, previous experience with nanomaterials was suggested to influence subjective norms (social pressure from others) leading to the intention to use nanomedicine [57].

## 6. Discussion

The present search aimed at providing a broad overview of clinicians’ perspectives toward the intention to use nanotechnological devices in neuro-oncology, with a focus on personal cognitive and affective factors that might affect the decision-making process.

With a focus on high-grade malignant brain tumors (e.g., glioblastoma), as to improving patients’ quality of life and life expectancy, new treatment strategies are worth being explored, but the readiness to introduce them in clinical practice should not be neglected. Insights into professionals’ attitudes toward the intention to use nanotechnology are poor, despite clinicians’ pivotal role in healthcare decision making. Theoretical frameworks have been proposed to give knowledge about laypeople’s and experts’ attitudes toward nanotechnology, but only a few studies have focused on clinicians’ perspectives.

Current models of risk assessment provide evidence of time-saving cognitive and affective shortcuts, i.e., heuristics to support people in the decision-making process under uncertainty, whereas they might be a source of bias. Whether the knowledge is poor, heuristics are more likely to occur.

Given the multi-professional nature of neuro-oncological care, where neurosurgeons, neurologists, oncologists, radiotherapists, and neuro-radiologists play a role, internal shared knowledge and perspectives are advisable, even more so if international consensus on treatment strategy is in the making. Multidisciplinary information exchange is recommended to provide personalized high-quality care, within both diagnostic and therapeutic pathways [59,60]. Discussion between professionals with different backgrounds should be encouraged to improve the understanding of each other’s knowledge and opinions toward a particularly important topic. Therefore, subjective perspectives have a twofold bearing since they might affect the willingness to adopt a new treatment approach at both individual and team levels. Furthermore, the head of the Clinical Unit, who is demanded to coordinate the workgroup, plays a pivotal role in decision-making, being able to shape the attitudes toward science and innovation in the healthcare structure itself.

Our search found no studies exploring clinicians’ perspectives towards nanotechnology within neuro-oncology, with neither qualitative nor quantitative assessment measurements.

Up-to-date perspectives on nanomedicine have been mostly investigated regardless of the field of application, despite research in its use spanning several medical domains and purposes, either for prevention or treatment. A more consistent search was retrieved under dental medicine [57,58] which suggested further investigation of the mutual interaction between socio-psychological factors and previous experience or knowledge of nanomedicine might be worthwhile to predict clinicians’ intention to use.

Self-report instruments with no theoretical framework were used to collect data, except in one study [58], where the Theory of Planned Behavior [61] was tested to predict the intention to use nanomaterials by dentists and dental hygienists. Sample sizes and participants’ characteristics were quite heterogeneous among studies, and enrollment was frequently extended to medical students. The unchanged nature over the last 10 years of clinicians’ knowledge and awareness of nanotechnology was remarkable, along with medical students raising concerns about proper university education in nanomedicine. Given the complexity and fast-growing nano-delivery systems in science, the need for new integrated curricula has recently been promoted in pharmacy programs [62]. Knowledge about current and future research in nanomedicine is worthy in medical education as well, so as to (i) provide reliable sources of information, such as classes, residency training, or scientific journals, (ii) enable proper decision making by upcoming healthcare workers, and (iii) minimize the influence of subjective variables. In addition, opinions and intentions to use nanotechnologies in clinical practice had been currently explored from the point of view of the individual, with no deepness in teamwork management.

In the current study, we identified a gap in the literature regarding clinicians’ perspectives on nanotechnology applications within glioblastoma. Hence, we decided to extend our research to applications within other medical fields to evaluate whether potential useful information can be transferred within glioblastoma research. Overall, the paucity of literature, methodological heterogeneity among studies, and the exploratory nature of our search itself did not allow us to conduct a more rigorous literature review. Thus, the present work does not claim to be exhaustive but rather encourages reflection on the current state-of-the-art of clinicians’ perspectives on nanomedicine in glioblastoma.

As our group of research is going to explore the intention to use a new experimental nanotechnological device for high-grade malignant glioma therapy, preliminary literature research on confidence in nanotechnology among healthcare workers is mandatory to build up a proper investigation. The possible impact of the use of a cancer cell trap device in the treatment of malignant gliomas through a dedicated survey directed to all specialists involved in the diagnosis and treatment of gliomas (neurologists, neurosurgeons, radiotherapists, oncologists, nuclear medicine physicians, neuroradiologists) is in the preparation stage. Therefore, future search might help to improve our knowledge of clinicians’ perspectives toward nanotechnology, with a focus on neuro-oncology.

## 7. Conclusions

Nanotechnology application in the medical field is growing fast. Biological barriers resulted in unsatisfactory therapeutics and put forward interest in innovative treatment approaches for glioblastoma. However, our findings suggest that knowledge from healthcare professionals might be poor. From a psychological perspective, if factual knowledge is weak, cognitive and/or affective heuristics are more likely to affect the risk of assessment and thus the individual attitudes and intention to use. Therefore, initiatives should be promoted to further engage clinical stakeholders in discussion. Moreover, an in-depth knowledge of nanotechnologies applied to glioblastoma beyond a technical-scientific perspective also accounting for psychological and ethical dimensions is needed.

## Figures and Tables

**Table 1:** Strengths and weaknesses of standard-of-care treatments.

Treatment	Strengths	Weaknesses
Newly diagnosed Glioblastoma		
Surgery	Enhance overall survival. Extent of resection is a prognostic factor.	Risk of new permanent neurologic deficits. Microscopic, finger-like growth of glioblastoma is imperceptible to presurgical or even intraoperative imaging techniques.
Radiation therapy (RT)	Improve the local control for the microscopic disease unaddressed by surgical resection.	Risk of radiation necrosis. Risk of neurotoxicity.
Temozolomide (TMZ) chemotherapy	The combination of RT and TMZ improves overall survival compared to radiation therapy alone (8% versus 1.9%).	THematologic toxicity (thrombocytopenia in 10–20% of patients). Non-hematologic toxicities: nausea, anorexia, fatigue, and hepatotoxicity. The advantage of combined treatment lasts for up to 5 years of follow-up. Activity is largely restricted to tumors with MGMT promoter methylation.
Tumor-treating fields (TTFields)	Improve progression-free and overall survival.	Unblinded nature and delayed time of randomization in trials. Cost, treatment compliance, and skin toxicity.
Bevacizumab	Longer progression-free survival.	Inconsistent effect on overall survival. Side effects: hypertension, thromboembolism, left ventricular dysfunction, proteinuria, delayed wound healing, and bleeding.
**Recurrent Glioblastoma**		
Further surgical resection	Improved survival advantage in selected patients.	Risk of new permanent neurologic deficits.
Re-irradiation	Better tumor control while maintaining the neurological status (especially in the form of stereotactic radiosurgery and fractionated stereotactic radiosurgery). Reduced use of steroids.	Risk of neurotoxicity. Limited prospective data.
Lomustine	Slight improvement in progression-free survival (1.5 months) [32].	Hematological toxicity. Activity is largely restricted to patients with tumors with MGMT promoter methylation.
Regorafenib	Encouraging overall survival benefit compared to Lomustine in randomized, controlled, phase II REGOMA trial.	A phase III trial is needed to confirm this benefit. Adverse events in REGOMA study: fatigue, hand–foot syndrome, lipase increase, bilirubin increase, and lymphocyte count decrease.

*Note.* From [33]

**Table 2:** Summary of studies investigating hospital workers’ attitudes and perceptions toward nanotechnology. For each study, the first author’s name, publication date, study design, recruitment setting, sample size, age range, job profile, theoretical framework, and aim are reported. The percentage of the subjects with previous knowledge or experience with nanomaterials/nanotechnology is reported, followed by assessment measure characteristics (type of measure and the number of items of the instrument).

First Author	Year	Country	Study Design	Recruitment Setting	Sample Size	Age	Job Profile	Previous Use or Literacy in Nanomaterials	Type of Measure	No. of Items	Theoretical Framework	Aim of the Study
Maldhure, S.	2021	India	Cross-sectional	Datta Meghe Medical College and Shalinitai Meghe Hospital	56	Not reported	Residents from the Departments of Medicine, Psychiatry, Dermatology, General Surgery, Orthopedics, Ophthalmology, ENT, Pediatrics and Obstetrics, Gynecology	14.3%	Self-administered questionnaire	8	Not reported	Collect information on awareness and knowledge about nanotechnology in upcoming clinicians.
Xenaki, V.	2019	Norway	Cross-sectional	Public Dental Health Service in Norway	791	22–70	Dentists and dental hygienists	46%	Self-administered questionnaire	20	Not reported	Assess whether socio-demographic factors, familiarity with nanotechnology, and social trust are associated with dental healthcare workers’ perceived risks and benefits of the use of nanomaterials in dentistry and whether those associations varied according to the professional status.
Xenaki, V.	2020	Norway	Cross-sectional	Public dental healthcare service	851	41.5 ± 11.9	Dentists and dental hygienists	54%	Self- administered an online questionnaire	28	Theory of Planned Behavior (TPB)	Predict the intention of dental healthcare workers to use nanomaterials in the future and explore whether the augmented TPB model operates equivalently across the professional groups of dentists and dental hygienists.
Nassani, N.	2019	USA	Cross-sectional	Staten IslandUniversity Hospital	70	29(range 25–35)	Postgraduate training internal medicine residents	8%	Self-administered questionnaire	35	Not reported	Evaluate the perception, knowledge, and attitude of medical residents toward nanomedicine.
Karthikeyan, H.	2018	India	Cross-sectional	Not reported	70	Not reported	Students in undergraduate and postgraduate teaching faculties and practitioners in dentistry and medicine	Not reported	Self- administered an online questionnaire	Not reported	Not reported	Create awareness of nanoparticles and their usage among professionals.
Ibrahim, N	2011	Saudi Arabia	Cross-sectional	Riyadh Military Hospital	300	18–60	Hospital employees and trainees	Not reported	Self-administered questionnaire	9	Not reported	Measure hospital workers’ awareness, perceptions, and preferences of nanotechnology and correlate them with existing demographic data.
Friedman, A. and Nasir, A.	2011	USA	Cross-sectional	Department of Dermatology	23	Not reported	Faculty and chief residents of dermatology training programs	30.4%	Self- administered an online questionnaire	21	Absent	Obtain specific information regarding dermatology knowledge, attitudes, and perceptions of nanotechnology.

## Data Availability

The data presented in this study are available on request from the corresponding author.

## Supplementary Materials

The following supporting information can be downloaded at: https://www.mdpi.com/article/10.3390/biomedicines11071854/s1. Table S1. Data extraction form. Table S2. Keyword included in the search query.

## References

[1] Omuro A., DeAngelis L.M. (2013). Glioblastoma and Other Malignant Gliomas: A Clinical Review. JAMA.

[2] Wirsching H.-G., Galanis E., Weller M., Berger M.S., Weller M. (2016). Chapter 23—Glioblastoma. Handbook of Clinical Neurology.

[3] Ostrom Q.T., Bauchet L., Davis F.G., Deltour I., Fisher J.L., Langer C.E., Pekmezci M., Schwartzbaum J.A., Turner M.C., Walsh K.M. (2014). The Epidemiology of Glioma in Adults: A “State of the Science” Review. Neuro-Oncol..

[4] Weller M., van den Bent M., Preusser M., Le Rhun E., Tonn J.C., Minniti G., Bendszus M., Balana C., Chinot O., Dirven L. (2021). EANO Guidelines on the Diagnosis and Treatment of Diffuse Gliomas of Adulthood. Nat. Rev. Clin. Oncol..

[5] Louis D.N., Perry A., Wesseling P., Brat D.J., Cree I.A., Figarella-Branger D., Hawkins C., Ng H.K., Pfister S.M., Reifenberger G. (2021). The 2021 WHO Classification of Tumors of the Central Nervous System: A Summary. Neuro-Oncol..

[6] Sarkaria J.N., Hu L.S., Parney I.F., Pafundi D.H., Brinkmann D.H., Laack N.N., Giannini C., Burns T.C., Kizilbash S.H., Laramy J.K. (2018). Is the Blood-Brain Barrier Really Disrupted in All Glioblastomas? A Critical Assessment of Existing Clinical Data. Neuro-Oncol..

[7] Mittal K.R., Pharasi N., Sarna B., Singh M., Rachana, Haider S., Singh S.K., Dua K., Jha S.K., Dey A. (2022). Nanotechnology-Based Drug Delivery for the Treatment of CNS Disorders. Transl. Neurosci..

[8] Manser T. (2009). Teamwork and Patient Safety in Dynamic Domains of Healthcare: A Review of the Literature. Acta Anaesthesiol. Scand..

[9] Sangaleti C., Schveitzer M.C., Peduzzi M., Zoboli E.L.C.P., Soares C.B. (2017). Experiences and Shared Meaning of Teamwork and Interprofessional Collaboration among Health Care Professionals in Primary Health Care Settings: A Systematic Review. JBI Evid. Synth..

[10] Schmutz J.B., Meier L.L., Manser T. (2019). How Effective Is Teamwork Really? The Relationship between Teamwork and Performance in Healthcare Teams: A Systematic Review and Meta-Analysis. BMJ Open.

[11] McGuier E.A., Aarons G.A., Byrne K.A., Campbell K.A., Keeshin B., Rothenberger S.D., Weingart L.R., Salas E., Kolko D.J. (2023). Associations between Teamwork and Implementation Outcomes in Multidisciplinary Cross-Sector Teams Implementing a Mental Health Screening and Referral Protocol. Implement. Sci. Commun..

[12] Batash R., Asna N., Schaffer P., Francis N., Schaffer M. (2017). Glioblastoma Multiforme, Diagnosis and Treatment; Recent Literature Review. Curr. Med. Chem..

[13] Nayak L., Lee E.Q., Wen P.Y. (2012). Epidemiology of Brain Metastases. Curr. Oncol. Rep..

[14] Ampie L., Woolf E.C., Dardis C. (2015). Immunotherapeutic Advancements for Glioblastoma. Front. Oncol..

[15] Colman H. (2020). Adult Gliomas. CONTINUUM Lifelong Learn. Neurol..

[16] Chaichana K.L., Jusue-Torres I., Navarro-Ramirez R., Raza S.M., Pascual-Gallego M., Ibrahim A., Hernandez-Hermann M., Gomez L., Ye X., Weingart J.D. (2014). Establishing Percent Resection and Residual Volume Thresholds Affecting Survival and Recurrence for Patients with Newly Diagnosed Intracranial Glioblastoma. Neuro-Oncol..

[17] Vivas-Buitrago T., Domingo R.A., Tripathi S., De Biase G., Brown D., Akinduro O.O., Ramos-Fresnedo A., Sabsevitz D.S., Bendok B.R., Sherman W. (2022). Influence of Supramarginal Resection on Survival Outcomes after Gross-Total Resection of IDH-Wild-Type Glioblastoma. J. Neurosurg..

[18] Guerrini F., Roca E., Spena G. (2022). Supramarginal Resection for Glioblastoma: It Is Time to Set Boundaries! A Critical Review on a Hot Topic. Brain Sci..

[19] Buatti J., Ryken T., Smith M., Sneed P., Suh J., Mehta M., Olson J. (2008). Radiation Therapy of Pathologically Confirmed Newly Diagnosed Glioblastoma in Adults. J. Neuro-Oncol..

[20] Ziu M., Kim B.Y.S., Jiang W., Ryken T., Olson J.J. (2020). The Role of Radiation Therapy in Treatment of Adults with Newly Diagnosed Glioblastoma Multiforme: A Systematic Review and Evidence-Based Clinical Practice Guideline Update. J. Neurooncol..

[21] Janjua T.I., Rewatkar P., Ahmed-Cox A., Saeed I., Mansfeld F.M., Kulshreshtha R., Kumeria T., Ziegler D.S., Kavallaris M., Mazzieri R. (2021). Frontiers in the Treatment of Glioblastoma: Past, Present and Emerging. Adv. Drug Deliv. Rev..

[22] Stupp R., Mason W.P., van den Bent M.J., Weller M., Fisher B., Taphoorn M.J.B., Belanger K., Brandes A.A., Marosi C., Bogdahn U. (2005). Radiotherapy plus Concomitant and Adjuvant Temozolomide for Glioblastoma. N. Engl. J. Med..

[23] Stupp R., Hegi M.E., Mason W.P., van den Bent M.J., Taphoorn M.J.B., Janzer R.C., Ludwin S.K., Allgeier A., Fisher B., Belanger K. (2009). Effects of Radiotherapy with Concomitant and Adjuvant Temozolomide versus Radiotherapy Alone on Survival in Glioblastoma in a Randomised Phase III Study: 5-Year Analysis of the EORTC-NCIC Trial. Lancet Oncol..

[24] Lukas R.V., Wainwright D.A., Ladomersky E., Sachdev S., Sonabend A.M., Stupp R. (2019). Newly Diagnosed Glioblastoma: A Review on Clinical Management. Oncology.

[25] Parisi S., Corsa P., Raguso A., Perrone A., Cossa S., Munafò T., Sanpaolo G., Donno E., Clemente M.A., Piombino M. (2015). Temozolomide and Radiotherapy versus Radiotherapy Alone in High Grade Gliomas: A Very Long Term Comparative Study and Literature Review. Biomed. Res. Int..

[26] Kaka N., Hafazalla K., Samawi H., Simpkin A., Perry J., Sahgal A., Das S. (2019). Progression-Free but No Overall Survival Benefit for Adult Patients with Bevacizumab Therapy for the Treatment of Newly Diagnosed Glioblastoma: A Systematic Review and Meta-Analysis. Cancers.

[27] Zhang S.-C., Hu Z.-Q., Long J.-H., Zhu G.-M., Wang Y., Jia Y., Zhou J., Ouyang Y., Zeng Z. (2019). Clinical Implications of Tumor-Infiltrating Immune Cells in Breast Cancer. J. Cancer.

[28] Tan A.C., Ashley D.M., López G.Y., Malinzak M., Friedman H.S., Khasraw M. (2020). Management of Glioblastoma: State of the Art and Future Directions. CA A Cancer J. Clin..

[29] Suchorska B., Weller M., Tabatabai G., Senft C., Hau P., Sabel M.C., Herrlinger U., Ketter R., Schlegel U., Marosi C. (2015). Complete Resection of Contrast-Enhancing Tumor Volume Is Associated with Improved Survival in Recurrent Glioblastoma—Results from the DIRECTOR Trial. Neuro-Oncol..

[30] Ryu S., Buatti J.M., Morris A., Kalkanis S.N., Ryken T.C., Olson J.J. (2014). The Role of Radiotherapy in the Management of Progressive Glioblastoma: A Systematic Review and Evidence-Based Clinical Practice Guideline. J. Neurooncol..

[31] Lombardi G., Salvo G.L.D., Brandes A.A., Eoli M., Rudà R., Faedi M., Lolli I., Pace A., Daniele B., Pasqualetti F. (2019). Regorafenib Compared with Lomustine in Patients with Relapsed Glioblastoma (REGOMA): A Multicentre, Open-Label, Randomised, Controlled, Phase 2 Trial. Lancet Oncol..

[32] Wick W., Gorlia T., Bendszus M., Taphoorn M., Sahm F., Harting I., Brandes A.A., Taal W., Domont J., Idbaih A. (2017). Lomustine and Bevacizumab in Progressive Glioblastoma. N. Engl. J. Med..

[33] Wu W., Klockow J.L., Zhang M., Lafortune F., Chang E., Jin L., Wu Y., Daldrup-Link H.E. (2021). Glioblastoma multiforme (GBM): An overview of current therapies and mechanisms of resistance. Pharmacol Res..

[34] Allan J., Belz S., Hoeveler A., Hugas M., Okuda H., Patri A., Rauscher H., Silva P., Slikker W., Sokull-Kluettgen B. (2021). Regulatory Landscape of Nanotechnology and Nanoplastics from a Global Perspective. Regul. Toxicol. Pharmacol..

[35] Schulte P.A., Geraci C.L., Murashov V., Kuempel E.D., Zumwalde R.D., Castranova V., Hoover M.D., Hodson L., Martinez K.F. (2014). Occupational Safety and Health Criteria for Responsible Development of Nanotechnology. J. Nanopart. Res..

[36] Slovic P., Fischhoff B., Lichtenstein S. (1982). Why Study Risk Perception?. Risk Anal..

[37] Fischoff B., Lichtenstein S. (1978). Don’t Attribute This to Reverend Bayes. Psychol. Bull..

[38] Tversky A., Kahneman D. (1974). Judgment under Uncertainty: Heuristics and Biases. Science.

[39] Kahneman D., Tversky A. (1979). Prospect Theory: An Analysis of Decision under Risk. Econometrica.

[40] Sjöberg L., Winroth E. (1986). Risk, Moral Value of Actions, and Mood. Scand. J. Psychol..

[41] Sjöberg L. (2007). Emotions and Risk Perception. Risk Manag..

[42] Brown V.J. (2014). Risk Perception: It’s Personal. Environ. Health Perspect..

[43] Lee C.-J., Scheufele D.A., Lewenstein B.V. (2005). Public Attitudes toward Emerging Technologies: Examining the Interactive Effects of Cognitions and Affect on Public Attitudes toward Nanotechnology. Sci. Commun..

[44] Cacciatore M.A., Scheufele D.A., Corley E. (2011). From Enabling Technology to Applications: The Evolution of Risk Perceptions about Nanotechnology. Public Underst. Sci..

[45] Ganesh Pillai R., Bezbaruah A.N. (2017). Perceptions and Attitude Effects on Nanotechnology Acceptance: An Exploratory Framework. J. Nanopart. Res..

[46] George S., Kaptan G., Lee J., Frewer L. (2014). Awareness on Adverse Effects of Nanotechnology Increases Negative Perception among Public: Survey Study from Singapore. J. Nanopart. Res..

[47] Kim Y.-R., Lee E.J., Park S.H., Kwon H.J., An S.S.A., Son S.W., Seo Y.R., Pie J.-E., Yoon M., Kim J.H. (2014). Interactive Survey of Consumer Awareness of Nanotechnologies and Nanoparticles in Consumer Products in South Korea. Int. J. Nanomed..

[48] Larsson S., Jansson M., Boholm Å. (2019). Expert Stakeholders’ Perception of Nanotechnology: Risk, Benefit, Knowledge, and Regulation. J. Nanopart. Res..

[49] Chaturvedi V.K., Singh A., Singh V.K., Singh M.P. (2019). Cancer Nanotechnology: A New Revolution for Cancer Diagnosis and Therapy. Curr. Drug Metab..

[50] Nasir A., Khan A., Li J., Naeem M., Khalil A.A.K., Khan K., Qasim M. (2021). Nanotechnology, A Tool for Diagnostics and Treatment of Cancer. Curr. Top. Med. Chem..

[51] Lam F.C., Salehi F., Kasper E.M. (2022). Integrating Nanotechnology in Neurosurgery, Neuroradiology, and Neuro-Oncology Practice-The Clinicians’ Perspective. Front. Bioeng. Biotechnol..

[52] Karthikeyan H., Vishnu Priya V., Gayathri R. (2018). Awareness on Use of Nanoparticles among Medical Professionals. Drug Invent. Today.

[53] Maldhure S., Sonwani V., Ambad R., Chandi D.H. (2021). Awareness about Nanotechnology among Upcoming Clinicians in Vidarbha, Maharashtra. J. Pharm. Res. Int..

[54] Ibrahim N. (2011). Hospital Workers Perceptions about Nano-Technology. Eur. J. Oncol. Pharm..

[55] Friedman A., Nasir A. (2011). Nanotechnology and Dermatology Education in the United States: Data from a Pilot Survey. J. Drugs Dermatol..

[56] Nassani N., El-Douaihy Y., Khotsyna Y., Shwe T., El-Sayegh S. (2020). Knowledge, Perceptions, and Attitudes of Medical Residents Towards Nanomedicine: Defining the Gap. Med. Sci. Educ..

[57] Xenaki V., Costea D.E., Marthinussen M.C., Cimpan M.R., Åstrøm A.N. (2020). Use of Nanomaterials in Dentistry: Covariates of Risk and Benefit Perceptions among Dentists and Dental Hygienists in Norway. Acta Odontol. Scand..

[58] Xenaki V., Marthinussen M.C., Costea D.E., Breivik K., Lie S.A., Cimpan M.R., Åstrøm A.N. (2021). Predicting Intention of Norwegian Dental Health-Care Workers to Use Nanomaterials: An Application of the Augmented Theory of Planned Behavior. Eur. J. Oral Sci..

[59] El Saghir N.S., Charara R.N., Kreidieh F.Y., Eaton V., Litvin K., Farhat R.A., Khoury K.E., Breidy J., Tamim H., Eid T.A. (2015). Global Practice and Efficiency of Multidisciplinary Tumor Boards: Results of an American Society of Clinical Oncology International Survey. JGO.

[60] Gaudino S., Giordano C., Magnani F., Cottonaro S., Infante A., Sabatino G., La Rocca G., Della Pepa G.M., D’Alessandris Q.G., Pallini R. (2022). Neuro-Oncology Multidisciplinary Tumor Board: The Point of View of the Neuroradiologist. J. Pers. Med..

[61] Ajzen I., Kuhl J., Beckmann J. (1985). From Intentions to Actions: A Theory of Planned Behavior. Action Control: From Cognition to Behavior.

[62] Barton A.E., Borchard G., Wacker M.G., Pastorin G., Saleem I.Y., Chaudary S., Elbayoumi T., Zhao Z., Flühmann B. (2022). Need for Expansion of Pharmacy Education Globally for the Growing Field of Nanomedicine. Pharmacy.

