# Supplementary material for: Insights into Healthcare Professionals’ Perceptions and Attitudes toward Nanotechnological Device Application: What Is the Current Situation in Glioblastoma Research?
Source: Biomedicines. 2023 Jun 28;11(7):1854. doi: 10.3390/biomedicines11071854 (PMC10376482; doi:10.3390/biomedicines11071854)
Supplement: Supplementary file 1 [file biomedicines-11-01854-s001.zip › biomedicines-2424087-supplementary.pdf]

Table S1. Data extraction form

Article	First author	Year	Country	Study design	Recruitment setting	Sample size	Age	Profile	Prior experience or education with nanomaterials (%)	Type of measure	Nr of items	Theoretic framework	Statistical anaylisis	Aim of the study

Article = article title

First Author = first author surname

Year = year of publication

Country = country where the study was conducted

Study design = study design

Recruitment setting = setting where participants were recruited (e.g. private/public hospital, university)

Sample size = participants sample size

Age = participants' mean age

Profile = participants' job profile (e.g. doctor – including field of specialization - , student, medical resident)

Prior experience or education with nanomaterials = percentage of responders reporting prior experience/education with nanomaterials

Type of measure = type of assessment measure (e.g. self-report)

Nr of items = number of items of the assessment instruments used to collect data

Theoretic framework = measures' reference model/theory

Statistical analysis = type of data analysis (e.g. descriptive, predictive, inferential)

Aim of the study = aim of the study

Table S2. Keyword included in the search query.

First keyword	*Second Keyword*	*Third Keyword*
Nanotechnolog*[Title/Abstract] OR	Physicians OR	Perception OR
nanomedicine[Title/Abstract] OR	Medical Residents OR	Attitude OR
Nanomedicine[Mesh] OR	Clinicians	Acceptance OR
Nanotechnology[Mesh]		Knowledge
